# Improving Inertial Pedestrian Dead-Reckoning by Detecting Unmodified Switched-on Lamps in Buildings

**DOI:** 10.3390/s140100731

**Published:** 2014-01-03

**Authors:** Antonio R. Jiménez, Francisco Zampella, Fernando Seco

**Affiliations:** Centre for Automation and Robotics (CAR), Consejo Superior de Investigaciones Cientificas (CSIC)-UPM, Ctra. Campo Real km 0.2, La Poveda, Arganda del Rey, Madrid 28500, Spain; E-Mails: francisco.zampella@csic.es (F.Z.); fernando.seco@csic.es (F.S.)

**Keywords:** indoor localization, signals of opportunity, light/illumination, pedestrian dead-reckoning, smartphone

## Abstract

This paper explores how inertial Pedestrian Dead-Reckoning (PDR) location systems can be improved with the use of a light sensor to measure the illumination gradients created when a person walks under ceiling-mounted unmodified indoor lights. The process of updating the inertial PDR estimates with the information provided by light detections is a new concept that we have named *Light-matching* (LM). The displacement and orientation change of a person obtained by inertial PDR is used by the LM method to accurately propagate the location hypothesis, and vice versa; the LM approach benefits the PDR approach by obtaining an absolute localization and reducing the PDR-alone drift. Even from an initially unknown location and orientation, whenever the person passes below a switched-on light spot, the location likelihood is iteratively updated until it potentially converges to a unimodal probability density function. The time to converge to a unimodal position hypothesis depends on the number of lights detected and the asymmetries/irregularities of the spatial distribution of lights. The proposed LM method does not require any intensity illumination calibration, just the pre-storage of the position and size of all lights in a building, irrespective of their current on/off state. This paper presents a detailed description of the light-matching concept, the implementation details of the LM-assisted PDR fusion scheme using a particle filter, and several simulated and experimental tests, using a light sensor-equipped Galaxy S3 smartphone and an external foot-mounted inertial sensor. The evaluation includes the LM-assisted PDR approach as well as the fusion with other signals of opportunity (WiFi, RFID, Magnetometers or Map-matching) in order to compare their contribution in obtaining high accuracy indoor localization. The integrated solution achieves a localization error lower than 1 m in most of the cases.

## 1. Introduction

Reliable and precise indoor localization of people is still an open problem, and many technological approaches have been proposed to achieve a usability similar to that obtained outdoors by the GPS system [1–3]. The most difficult challenge for pedestrian navigation is to find an accurate-enough indoor location method, valid for extended areas, which can withstand environmental condition changes, and at the same time is as simple as possible. Two main approaches can be used for the location of persons indoors: (1) Solutions that rely on the existence of a network of receivers or emitters placed at known locations (beacon-based solutions or Local Positioning Systems-LPS) [4–6]; and (2) Solutions that mainly rely on dead-reckoning methods with sensors only installed on the person to be located (beacon-free solutions, or Pedestrian Dead Reckoning-PDR) [7–10]. The current tendency is the hybridization of both approaches [11,12].

The use of *signals of opportunity* for the localization of persons indoors is a recent and very promising approach, especially from the usability point of view. Signals of opportunity are those not originally meant for localization purposes but which are freely available most of the time in standard spaces, without requiring the installation of any ad-hoc infrastructure. Some common signals of opportunity are: telephony, FM/TV broadcast, WiFi, Bluetooth, magnetic fingerprints, illumination, pressure, temperature, among others [13,14]. A modern smartphone is a convenient device to register most of these signals.

One of those signals of opportunity is the *light intensity* from standard light sources. The estimation of the position of a person indoors using unmodified artificial lights (e.g., fluorescent lights) has received little attention in the research community. A few authors [15–18] used unmodified light signals to perform room-level localization using fingerprinting approaches to infer in which room the user could be located. They assume that the illumination intensity and/or ambient color is different among particular rooms. This approach has the limitations of providing just a symbolic positioning (*i.e.*, poor physical location accuracy), and the necessity to perform frequent re-calibrations to update the measurement models, since lighting conditions can change.

A very innovative approach has been studied at the Wearable Computing Laboratory (ETH Zürich) [19–21], proposing the use of light intensity variations from standard fluorescent lights to estimate the relative displacement of a person while walking. The estimation is based on how the sensed illumination changes as the distance from the user to the light source varies. The user has a small solar panel, embedded on the clothes, that is used as an illumination sensor. Although this approach has a great merit and obtains good results in some short-range experiments (aprox. 0.2 m distance error for a 8-meter-long path [19]), it has some factors that limit the achievable accuracy in estimating the displacement of the person: illuminance depends on the orientation of the receiver, influence of close-by windows with natural light, sensitivity to the light rated power (different behavior e.g., for 30 or 60 Watts lights), influence of aging of lights, dust accumulation, influence of the reflectivity of surrounding objects, diffusers, *etc.* Additionally, as the method estimates the relative traveled distance, it has to be integrated with an absolute positioning system (RFID-based in this case) in order to be able to estimate the user location.

In [22], a robot moving in an indoor space is able to detect unmodified light spots with a camera pointing to the ceiling. The robot estimates its pose using wheel-based odometry, and occasionally the robot's pose estimation is corrected when a light is detected. The approach of fusing dead-reckoning measurements with light detections is very interesting, and that basic idea will be followed in our paper with several improvements and added value. One of the weaknesses of the system proposed in [22] is the assumption that only one light pattern can be recorded in an image in order to avoid the misidentification of lamps, as well as the assumption of an initially known robot position. As they start with a valid pose estimate, their method makes positioning corrections with the closest lamp of a previously “learned” lamp database. This limited operational mode of correcting position using only the estimated closest lamp results from a method which does not support multiple hypothesis and therefore it is not robust against false light detections or unknown initial position information.

*Light-communication* [23,24] is another approach for absolute indoor localization using lights which are modified by adding electronic current modulators. In this manner each particular light emits or “communicates” a unique identification, or alternatively, its position. This is a similar concept to infrastructure-based LPS localization, and it is far from the unmodified approaches in [15–22] cited above, or the LM approach proposed in this paper.

**Figure 1.**
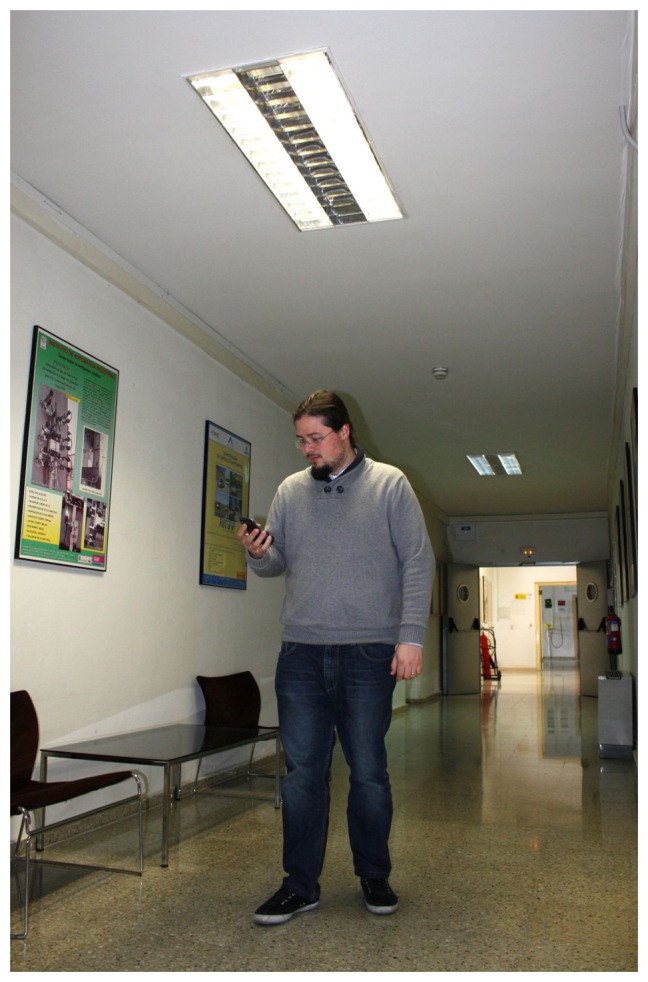
Fine-grained location of people indoors using the *Light-Matching* approach, in which the location likelihood of a person is updated when he passes under an (unmodified) light spot holding a smartphone. Inertial-based Pedestrian Dead-Reckoning (PDR) is used as a motion model to propagate his location likelihood. Other available sensors (magnetometer), context information (map) or signal of opportunity (WiFi) can be used as a backup or to improve/guarantee a unimodal location estimation.

In this paper we introduced the concept termed *Light-matching*, which is a new way to achieve accurate physical location, complementing the displacement and rotational information provided by inertial PDR methods with the information obtained by detecting unmodified lights in indoor environments (see Figure 1). The lights are detected by analyzing the illumination gradient that is generated when a person walks under a switched-on lamp. As in other matching techniques, we need to know the 2D position, size and orientation of all ceiling-mounted lamps in a building, however the current lighting state (if they are on or off) is not needed. Even from an initially unknown location and orientation, whenever the person passes below an switched-on light spot, the location likelihood is iteratively updated until the likelihood potentially converges to a unimodal probability density function. The time to converge to a unimodal position hypothesis depends on the number of lights detected and the asymmetries/irregularities of light distributions. This PDR + LM fusion approach can be used in cooperation with other signals of opportunity (WiFi, Magnetometers or map-matching) to obtain even better indoor localization accuracy.

This paper presents a significant extension to the basic concept already made available in a communication [25] by the authors of this work. New contributions include the analysis of how multiple hypothesis can be pruned using three common sources of information: (1) magnetic fields; (2) the existence of irregular light distributions; and (3) the cooperation with other sensor information (WiFi, RFID and GPS). The description of the light detection process as well as the Bayesian modeling for each lamp are also extended. We also go beyond the simulated evaluations and in this paper we incorporate an on-the-field experimental validation of the concept using a Smartphone to collect all the required information (Illuminance, Inertial, WiFi, GPS, RFID, and so on).

The paper presents the extended description of the Light-Matching concept in Section 2, the implementation details in Section 3, and several simulated and experimental tests using the smartphone in Sections 4 and 5 respectively. Finally, in the last two sections, we provide a discussion about the benefits and limitations of this approach, future work to be done, and some final conclusions.

## 2. Light-Matching Concept

This section explains the basic Light-Matching idea, *i.e.*, how to use unmodified lights to determine the user's location assuming that Dead-Reckoning (DR) information is also available. We also analyze the localization convergence, measured as the change in the number of location hypothesis, and how it is influenced by the number of lights in a building and the number of detections. Then, we explain how a typical non-regular distribution of lights in a building can have significant benefits in the localization process, as well as, the interest of aiding the system with an electronic compass.

### 2.1. Basic Light-Matching Idea

The Light-Matching idea is very simple. If we know exactly where all the lights in a building are located (2D position, size and orientation of each light in each floor), and we assume that we are able to detect when a person has passed under a light spot, then if a light is detected we can infer that the person is located under some of the lamps in the building. So, under an unknown initial position and orientation, a light detection implies as many local hypothesis as the number of lights in a building. These multi-modal location hypotheses are propagated according to a motion model that can be estimated with pedestrian dead-reckoning techniques. After a second light detection, the current multi-modal hypothesis located under any light spot will persist, and the rest of the hypothesis will be discarded. This process of hypothesis propagation (motion model) and hypothesis update at light detections (measurement model), finally can converge to a unimodal hypothesis that represents the true location of the person. This concept can be straightforward implemented using a Bayesian approach with a particle filter technique, in which the state *X* of each particle consists in the location and orientation of the person (*X* = {*x*, *y*, *z*, *θ*}).

**Figure 2.**
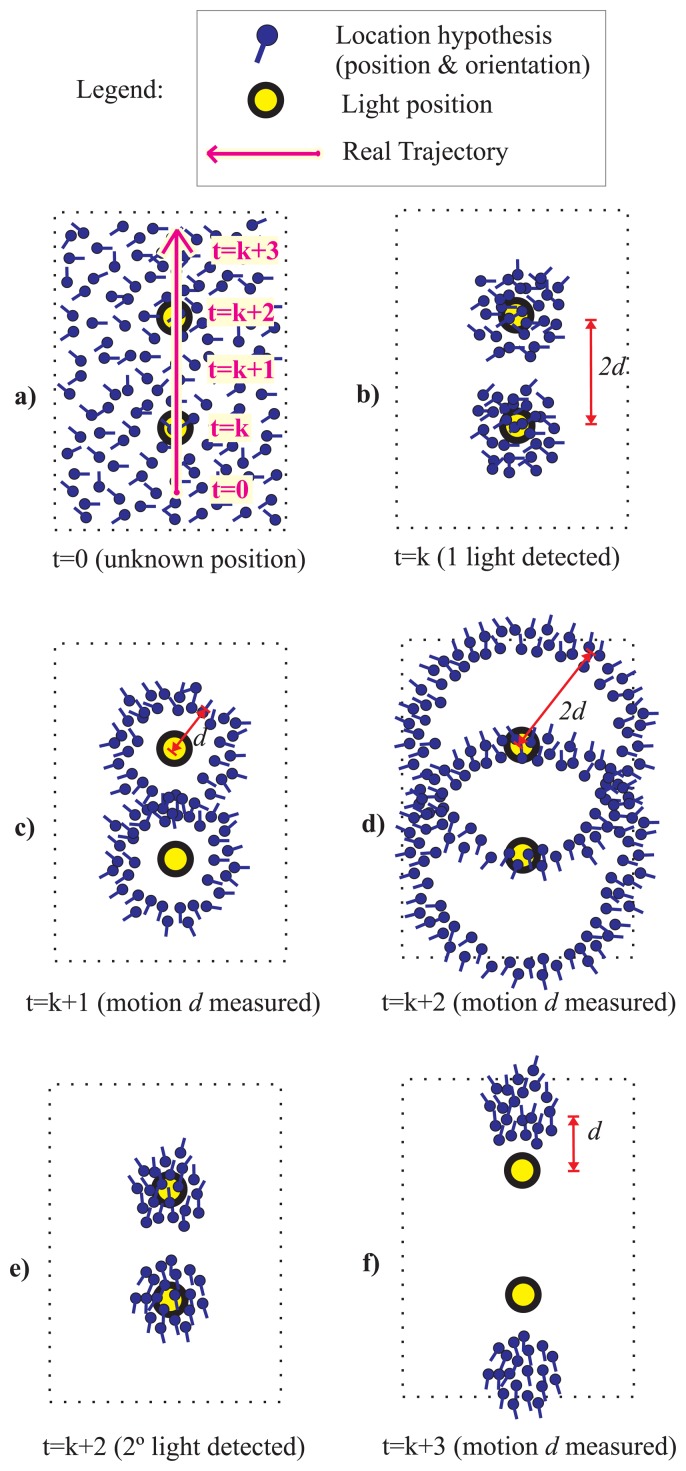
Light-Matching concept applied to estimate the location and heading of a person. Example of how the uncertainty in the estimation is reduced when the person gets under two lights while walking straight in an indoor area. (**a**) Initially (t = 0) there is no information about the location of the person in the area (represented by multiple location hypothesis at several places and with different orientations); (**b**) The person is under a light, so he must be at any of the light locations; (**c**) the persons moves a distance *d* since last light detection (measured by PDR); (**d**,**e**) after moving straight a distance d at time t = k + 2, the person is under another light; (**f**) After this second light detection there are only two main cluster of location hypothesis with well-defined position and orientation (one of those corresponds to the true location).

In Figure 2 the Light-Matching concept is explained with an example. We illustrate the case of a person that is walking straight (magenta arrow) in a simplified building floor with only two light spots (yellow circles) separated by a distance *2d*. We use several particles to represent the likelihood of the location hypothesis, as well as their orientations (represented by a short stem at each particle). At the beginning (t = 0), Figure 2a, we do not know where the person is located, so the particles are spread in the floor-plan with a uniform orientation distribution. When the user moves under a light and detects it for the first time (t = k), then we know that he can only be under any of the two lights in the building. This is represented by a double cluster of particles that represent two local unimodal hypothesis (Figure 2b). Note that these two hypotheses have not any preferred orientation (*i.e.*, uniform distribution). While the user walks straight a distance d, at time t = k + 1 (Figure 2c), and another distance d at t = k + 2, the particles separate from the light centers according to their individual orientation creating a circular ring for each light position (Figure 2d). At this time (t = k + 2) a second light is detected, which causes an update that reinforce the likelihood under the two lights and eliminates the remaining particles (Figure 2e). Note that this two hypothesis have, apart from distinctive locations, two defined orientations (particles at upper cluster point “North”, and those at the lower cluster point “South”). If the user continues his straight motion both clusters are moved according to the PDR estimation. We know that the upper hypothesis is the correct one (we advanced the real trajectory in Figure 2a) but without any prior knowledge both hypothesis are equally valid, one that corresponds to a straight path from South to North starting below, and another from North to South starting above in the floor map.

### 2.2. The Total Number of Hypotheses: Influence of the Number of Lights and Detections

In this paper we are using the term “*hypothesis*” to refer to any unimodal location likelihood or equivalently, to any cluster of particles that share a similar position and orientation. Each particle itself, although it is also an hypothesis, only represents a sample of the probability density function of the overall distribution.

In the example of the previous subsection (Section 2.1 and Figure 2), consisting of two lights in a floor, we found that after passing under two lights the number of *hypothesis* was two, *i.e.*, equal to the number of lights in the space. In general, if the number of lights in the building, *n*_lights_, increases it is expected that the number of hypotheses, *n*_hyp_, will grow proportionally after the first light detection. So, *n*_hyp_ ∝ *n*_lights_ for only one light detection. If the person moves, from that location, the particles in each hypothesis will be spread forming rings of particles centered in each light position (as in Figure 2c,d). If we consider that we can split each ring of particles in several quadrants, for example 4 or 8 quadrants, corresponding to angle ranges of 90 or 45 degrees, respectively, then we can consider that each ring of particles contains *n*_quad_ hypothesis (being for example *n*_quad_ = 8). So, in general we can say that the number of hypotheses after just one light detection is equal to the number of light multiplied by the number of quadrants, *i.e.*, *n*_hyp_ = *n*_lights_ · *n*_quad_.

The growth of the number of hypotheses with the numbers of lights in a building, which is an undesirable feature, however can be alleviated by the number of light detections (*n*_det_) that occur while the person is walking indoors. In Table 1 it is shown the systematic calculation of the total number of hypotheses for a different number of aligned lights installed in a corridor in a building, and for different number of detections while a person is walking straight in each case. We see that when the number of detections is larger that two, then the number of hypotheses begins to diminish proportionally. The following formula can be generalized for this series of data:

(1) $$n_{\text{hyp}}=\{\begin{array}{ll} 2\cdot (n_{\text{light}}+1)-2\cdot n_{\det} & ifn_{\det}\geq 2\\ n_{\text{light}}\cdot n_{\text{quad}} & ifn_{\det}=2\\ \infty & ifn_{\det}=0 \end{array}$$

which although is only valid for the case of a person moving straight, it gives us a concrete idea of how the number of hypotheses depend on the number of lights in the building and the number of light detections. From this case, we can see that visiting half of the lights in the building (*n*_det_ = *n_l_*_ights_/2) the number of hypotheses is similar to the number of lights (*n*_hyp_ = *n*_lights_ + 2). Even visiting all the lights in a building (*n*_det_ = *n*_lights_) the minimum number of hypotheses is still 2 (*n*_hyp_ = 2). This means that additional methods to prune the hypothesis are needed.

### 2.3. Pruning the Number of Hypotheses

We have seen that one of the drawbacks of the Light-matching concept is that it generates multiple location hypotheses (proportional to the number of lights, Equation (1)). Nevertheless, the Light-matching approach can frequently converge to a unique location hypothesis under one or several of the following common circumstances:

- Using the Earth's magnetic field.
- Existence of Irregular Light Distributions.
- In cooperation with other sensors/available information.

Next we will explain how these common circumstances help to prune the number of hypotheses.

#### 2.3.1. Using the Earth's Magnetic Field

Estimating the spatial orientation of a person in indoor environments by measuring the Earth's magnetic field is not, in general, too reliable due to typical magnetic perturbations (metallic structures, motors, computer equipment, *etc.*). Many authors have avoided the use of magnetometers for heading estimation [26,27], however others have characterized statistically the magnetic perturbations on the heading angle and have used the information in positioning applications [28–31]. Some of these papers report standard deviations of the errors in heading of 0.68 radians in difficult buildings made of metallic frames, and an approximate Gaussian distribution with a close to zero mean.

**Figure 3.**
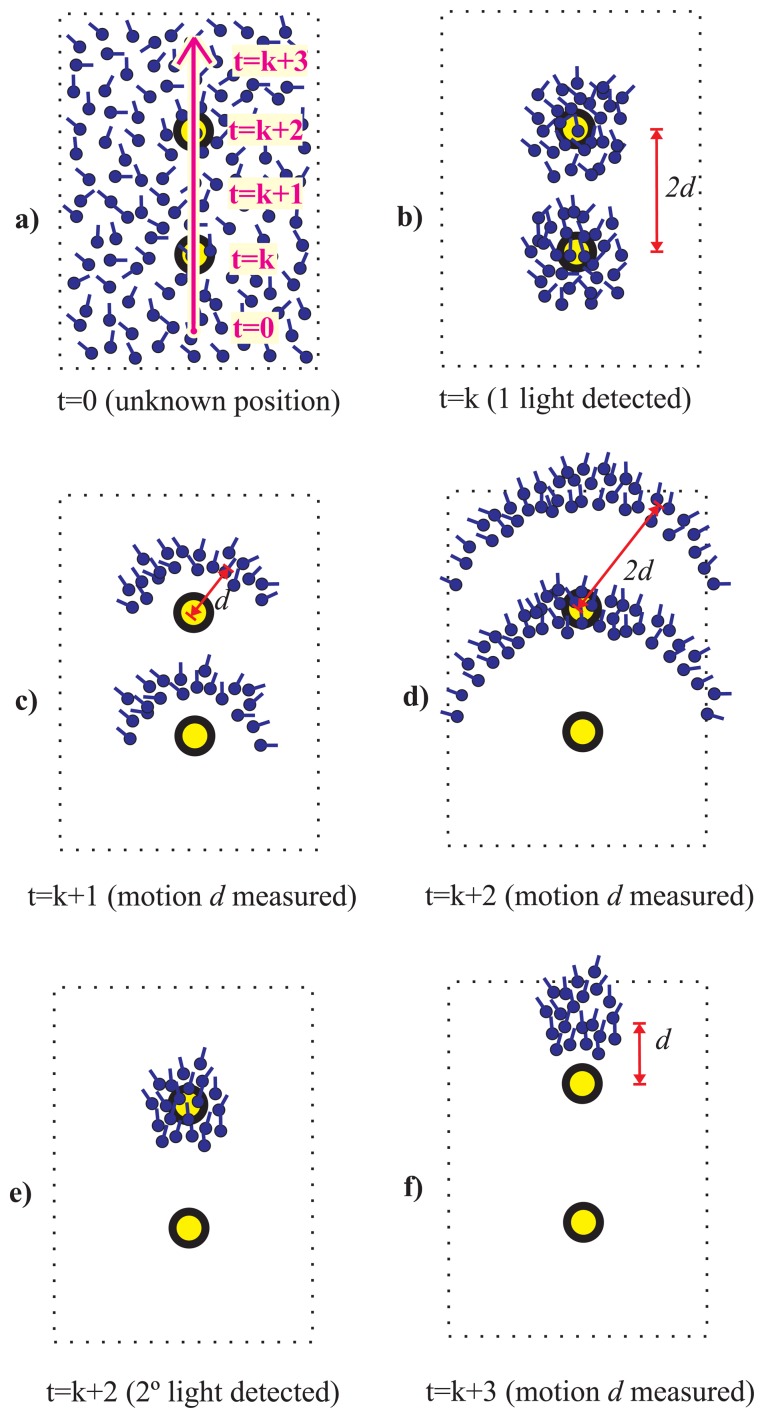
Light-Matching concept improved by a gross **electronic compass** indication. Same cases as in Figure 2. Now there is only one cluster hypothesis, after the double light detection, representing the true location and heading of the pedestrian.

We state that using an appropriate magnetically-based orientation model it is possible to prune some of the hypotheses in the Light-matching method. In particular, even using a very pessimistic model that assumes large magnetometer error, it could be very easy to prune those hypotheses whose orientations are in the opposite direction to the real one. See the example in Figure 3, which is similar to the one in Figure 2, but in this case we use information from an electronic compass modeled with a standard deviation error larger than one radian. It is clear how after detecting two lights only one hypothesis holds.

In general, for a 2D light distribution in a floor, and assuming that with the magnetometer we can differentiate between 4 quadrants (angles *π*/2 apart) the number of hypotheses can be reduced by two as compared to the case of not using a compass. This reduction can be further improved with the next situations.

#### 2.3.2. Benefits from Irregular Light Distributions

An irregular distribution of lights in a building causes that the distance among near-by lights are not always *d* or $d\sqrt{2}$. This fact helps to prune the number of hypotheses quickly. Although it may be thought that regular light distributions are quite common in the entire building floor, in practice this is not so usual because of the following reasons:

- *Light densities are not identical in different rooms.* The number of lights per unit area, or light density, is different among buildings. It depends on the type of lamps, their directionality and intensity, even the kind of activity that is performed there and the height of the ceiling. When the density, or inter-lamp separation, is different at different rooms, then the movement of the user from a room to another can help to prune the number of hypothesis.
- *The reference frame to install lights is different from room to room.* There can be a positioning shift of the reference frame between rooms. This can be possible even for regular distributed lights within a room having inter-light distances that are multiples of *d* or $d\sqrt{2}$. However, even in that case, the distance between the lights of different rooms does not have to be a multiple of *d* or $d\sqrt{2}$. This fact also causes hypothesis pruning when the user makes transitions from room to room.
- *There are light asymmetries in pathways.* There are common pathways such as corridor with typical L-shape or U-shape configurations, that makes lights distributions to be asymmetrical. This fact also causes hypothesis pruning when the user travels along corridors, specially at corners or turns.

**Figure 4.**
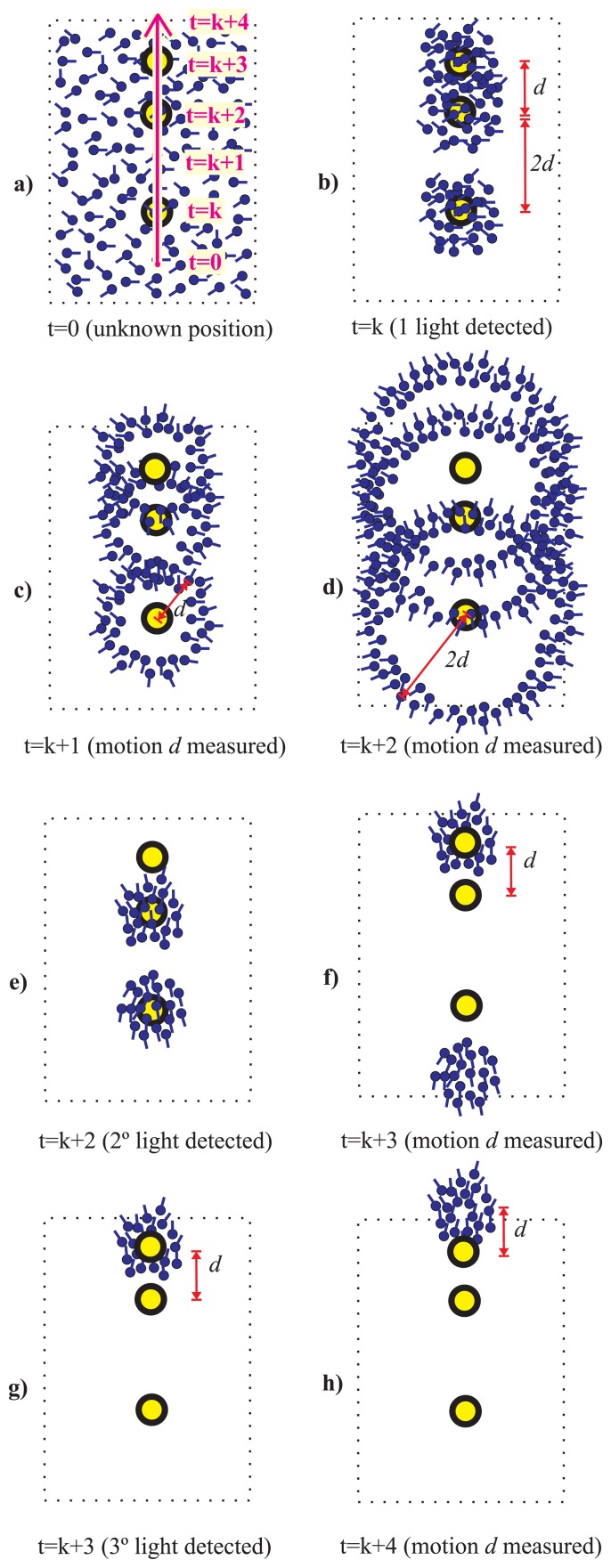
Light-Matching concept improved by **irregular distributions of lights**. Same case as in Figure 2. After the third light detection, there is only one cluster hypothesis, representing the true location and heading of the pedestrian.

A simple example of an irregular light distribution (different density) can be seen in Figure 4, where three lights are aligned but two of them are separated by a distance *d* and the other pair by a distance 2*d.* After the third light detection only one hypothesis persists, even without use of a magnetometer.

**Figure 5.**
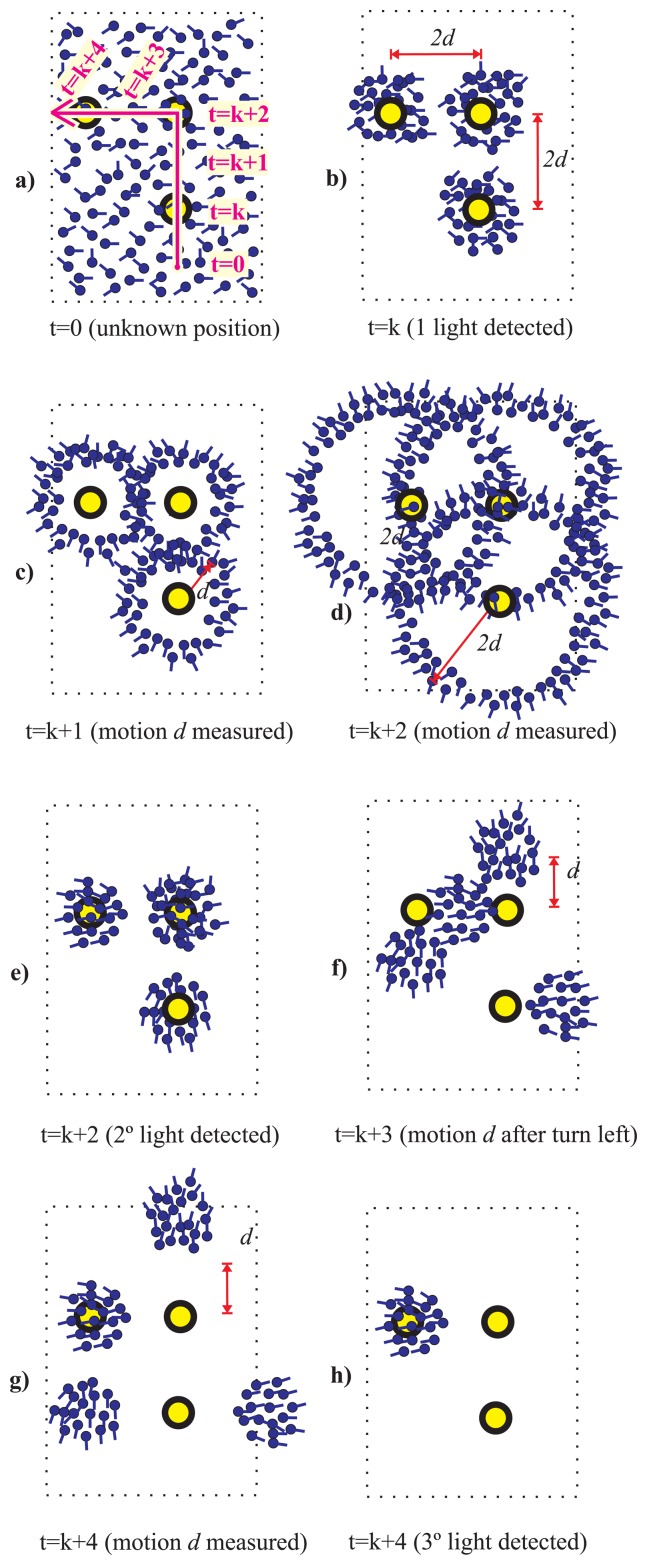
Light-Matching concept improved by **asymmetric distributions of lights**. Same cases as in Figure 2. Now there is only one cluster hypothesis, after the third light detection, representing the true location and heading of the pedestrian.

Another example to illustrate the effect of light asymmetries is presented in Figure 5. In this case three lights are distributed regularly with a distance of 2*d* and $2d\sqrt{2}$ among them. However the grid is not complete, since this situation could correspond to a corridor with L-shape. At the second light detection, it is also detected a turn of the person to the left, therefore hypothesis are translated and rotated accordingly. When the third light is detected only one hypothesis is conserved.

#### 2.3.3. Benefits from other Information and Signals of Opportunity

The above presented Light-matching concept, which includes the light detections, the measurement of displacements and turns with PDR techniques, and the pruning of hypotheses by gross magnetic information and by the natural irregularities/asymmetries of lights in a building, represents the basic Light-matching approach. This approach can converge in many situations, but this fact is not guaranteed, so any additional information available from the unmodified environment is beneficial.

Map-matching with a preloaded indoor map can be used to prune most of the particle or hypotheses that try to cross any of the walls in the building. This method of localization, very usual in indoor robotics literature as well as for pedestrians [32–35] is a good approach to guarantee the convergence to a unimodal likelihood and to speed the convergence up. Combining Light-matching and Map-matching is mutually beneficial since neither approach guarantees convergence when there are symmetries. However, when fusing both methods the probability of convergence is much higher since the symmetries in the wall map are not necessarily correlated with the light distributions.

Of course, any additional signal of opportunity coming from preexisting or unmodified infrastructure, such as for example, the Received Signal Strength (RSS) of WiFi access points, could be used to prune further the set of potential location hypothesis. The advantage of this integrated approach is that the density of the beacons generating these signals of opportunity does not have to be as high as in positioning solutions that rely only in RF fingerprinting or RF-trilateration. As a rule of thumb, we estimate that using the RSS of one of these natural beacons (WiFi AP) could potentially reduce the initially high number of hypotheses to approximately one half in a short period of time. We will return to this aspect during the evaluation stage (Section 4).

## 3. Light-Matching Implementation in a Pedestrian Localization Framework

**Figure 6.**
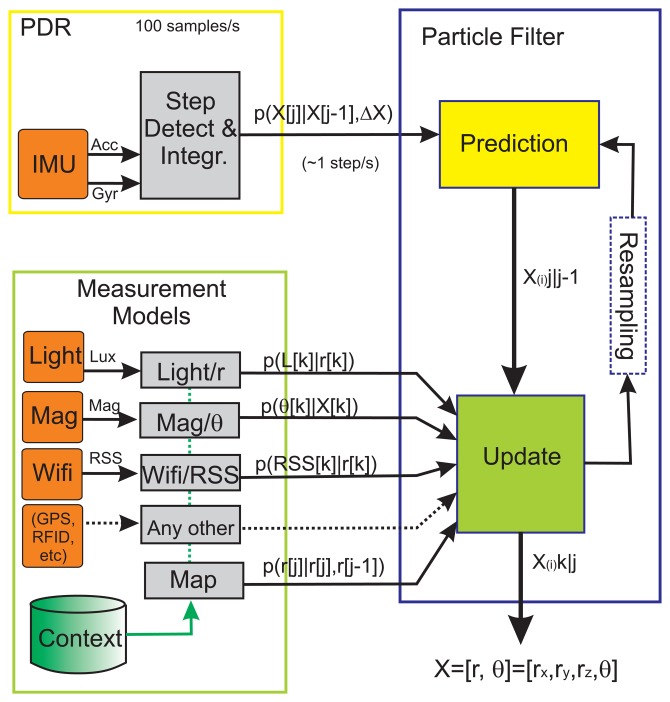
General Pedestrian Localization Framework based on a Particle Filter implementation. This approach is used in this paper to include the light detections and the Light-matching measurement model.

In this section we give the implementation details of our localization methodology. We use a Bayesian filter, implemented with a Particle Filter (PF), to integrate the measurements coming from the light detections, as well as any other information that could be available (Magnetometer, Wifi, GPS, Map, *etc.*). The motion information is provided by a PDR subsystem that is also integrated in the same framework (see Figure 6).

### 3.1. Particle Filter-Based Pedestrian Localization Framework

In our PF implementation we use a *state vector*, *X*, composed of 4 components: *X* = {*r_x_*, *r_y_*, *r_z_*, *θ*}. The first three terms represent the 3D position, *r* = {*r_x_*, *r_y_*, *r_z_*}, and the last term *θ* is the heading with respect an arbitrary-selected local navigation frame. We use a classical recursive *prediction* of the state vector, followed by an *update* of the state when a measurement is available. Occasional *resamplings* are performed when a degeneration of the particles is detected [36]. Next it is detailed each of these prediction and update stages.

#### 3.1.1. PDR-Based Prediction

**Figure 7.**
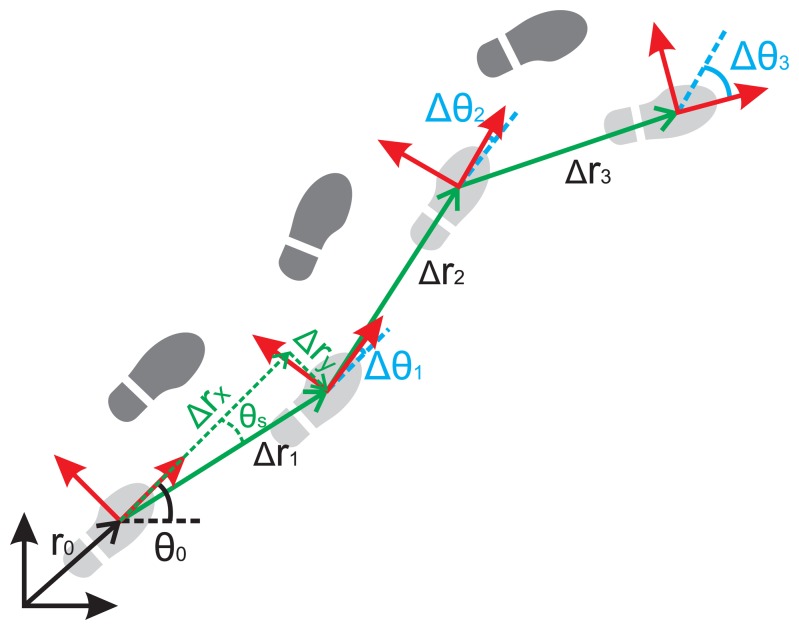
Reconstruction of the position using the step displacements (green) and heading changes (blue) obtained from PDR and starting from the initial position *r*_0_ and heading *θ*_0_.

A *prediction* is performed whenever a step *j* is detected with the PDR subsystem. This PDR module estimates the PDR inter-step changes, Δ*X*[*j*] = {Δ*r_x_*[*j*], Δ*r_y_*[*j*], Δ*r_z_*[*j*], Δ*θ*[*j*]}, from the last step *j* − 1 to the current step *j*, using a Kalman-based INS algorithm with ZUPT updates [37]. We use a foot-mounted INS integration method because it is possible to accurately estimate the changes in the 3D position and the foot heading with respect to the last step pose. In Figure 7 these inter-step changes are depicted, as well as the trajectory of the right foot of a pedestrian after some step detections.

The prediction stage in the PF moves all the particles, *X*^(^*^i^*^)^, for *i* = 1… *N*, from the last detected step at time *t*_PDR_[*j* − 1], according to the estimated ΔX[*j*] of the current step, at time *t*_PDR_[*j*]. This propagation (movement and rotation) of the particles states also includes the addition of some random state values, that represent the uncertainty of the movement model [37,38], *i.e.*,

(2) $$X^{\left(i\right)}[j]=X^{\left(i\right)}[j-1]+f(\Delta X[j],n_{\text{step}},\theta^{\left(i\right)}[j-1])$$

where *n_step_* ∼ 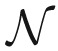 (0, P[*j*]), *i.e.*, represents the covariance error model of the PDR prediction. As the inter-step changes are always measured in the reference frame of the previous step, it is needed to transform the Δ*X* estimations to the localization frame. The function *f* includes the non-linear Z-axis rotation operation by *θ*^(^*^i^*^)^ [*j* − 1] to transform the Δ*X* PDR estimations, referenced in the frame of the last step detection (*j* − 1), to the local reference frame.

(3) $$f(\Delta X[j],n_{\text{step}},\theta^{\left(i\right)}[j-1])=\left[\begin{array}{cccc} \cos(\theta^{\left(i\right)}[j-1]) & -\sin(\theta^{\left(i\right)}[j-1]) & 0 & 0\\ \sin(\theta^{\left(i\right)}[j-1]) & \cos(\theta^{\left(i\right)}[j-1]) & 0 & 0\\ 0 & 0 & 1 & 0\\ 0 & 0 & 0 & 1 \end{array}\right]\times \left(\begin{array}{cc} \left[\begin{array}{c} \Delta r_{x}[j]\\ \Delta r_{y}[j]\\ \Delta r_{z}[j]\\ \Delta \theta [j] \end{array}\right] & +\sqrt{P[j]}\cdot {\text{randn}}^{\left(i\right)}(4,1) \end{array}\right)$$

where “randn^(^*^i^*^)^ (4,1)” is 4-value column of normally distributed pseudorandom numbers.

#### 3.1.2. Measurement-Based Update

The *update* stage to refine the predicted state of the particles is computed whenever a measurement *k* is received at time t_meas_[*k*]. Note the different notation (*j* and *k*) to represent the index of steps and measurement occurrences, respectively, which in general occur at different time instants (*t*_PDR_[*j*] and *t*_meas_[*k*]). A different measurement model exists for each type of measurement (Light-matching, magnetometer, Wifi, Map-matching, *etc.*). According to these models the weight of each particle, representing the likelihood of the user being at a certain position and orientation, is changed:

(4) $$w^{\left(i\right)}[k]=w^{\left(i\right)}[k-1]\cdot p(z[k]|{\hat{X}}^{\left(i\right)}[k])\cdot \alpha$$

where *p*(*z*[*k*]|*X̂*^(^*^i^*^)^[*k*]) is the likelihood function obtained from the measurement *z*[*k*] when the state of the particles at time t_meas_[*k*] is estimated to be *X̂*^(^*^i^*^)^ [*k*]. The term α is a normalization factor to guarantee that the sum of all probabilities is equal to 1.

Note that as the state of the particles is only sampled at step detections (*i.e.*, at times: *t*_PDR_[*j*] for *j* = 1… Num_steps_), then we normally must extrapolate the particles' state at times of measurement (*t*_meas_[*k*]), in order to obtain *X̂*[*k*] and to apply the measurement correction on-line, *i.e.*, at the instant when the measurement is received. So *X̂*[*k*] is an approximation of the position and orientation of the particles assuming a constant velocity between the last two step points, as:

(5) $${\hat{X}}^{\left(i\right)}[k]=X^{\left(i\right)}[j-1]+\gamma \cdot (X^{\left(i\right)}[j]-X^{\left(i\right)}[j-1])$$

where *γ* is the extrapolation weight:

(6) $$\gamma =\frac{\left(t_{\text{meas}}[k]-t_{\text{PDR}}[j-1]\right)}{\left(t_{\text{PDR}}[j]-t_{\text{PDR}}[j-1]\right)}$$

Finally, the output of the filter, which is computed at the step detection rate (aprox. 1 Hz), uses the states for the last detected step, $X^{\left(i\right)}[j]=\{r_{x}^{\left(i\right)}[j],r_{y}^{\left(i\right)}[j],r_{z}^{\left(i\right)}[j],\theta^{\left(i\right)}[j]\}$, and the weights updated with the last measurement, *w*^(^*^i^*^)^ [*k*], to estimate the localization and orientation of the user:

(7) $$X_{\left(j,k\right)}=\sum_{i=1}^{N}X^{\left(i\right)}[j]\cdot w^{\left(i\right)}[k]$$

### 3.2. Light-Matching Measuring Components

**Figure 8.**
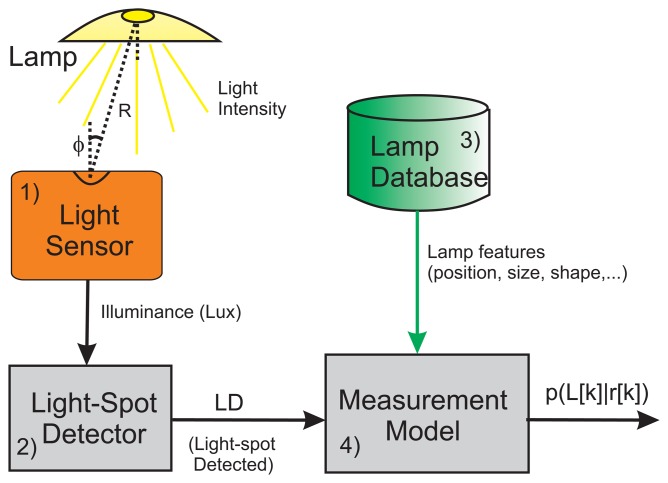
Components in the measurement subsystem of the Light-matching approach: (1) Illumination sensor; (2) Light spot detector; (3) Lamp database; and (4) Measurement model.

The measurement subsystem of the Light-matching approach consists basically of 4 components: (1) a *sensor* to capture the ambient illumination; (2) a *light spot detector*, which is a signal processing block to analyze illumination changes in order to deduce when a person has walked under a light; (3) a *database* including the coordinates and features of all lamps in a building; and (4) a *measurement model*, *p*(*L*[*k*]|*r*[*k*]), that represents the probability of detecting a light while the user is at a certain location *r* at time *t*_meas_[*k*]. This components are depicted in Figure 8, and explained in more detail next.

#### 3.2.1. Illumination at the Sensor

There are different illumination technologies for indoor use, such as, incandescent lights (tungsten bulbs, halogen), discharge lamps (fluorescent, Xenon,…), and LED lamps. The Light-matching concept is independent of the type of technology employed; the only requirement is that when approaching/passing under a lamp the illumination captured by the sensor should change. This fact is in general true for most lights, for a source of light without reflections, the irradiance *E* (W/m^2^) or illumination (lumen/m^2^ = lux) over a surface at a distance *R* from a lamp, follows the inverse square distance law, *i.e.*, *E* ∼ *I*/*R*^2^, where *I* is the radiant intensity (lumen/sr = candela) that emits a source of light. In a typical configuration, with lamps on the ceiling, and a person walking along a horizontal surface (the floor), the distance *R* between the user and the lamp reaches a minimum (the illumination is higher) when the user is under the lamp. The change in the registered illuminance is used to detect the light spot, as explained later in next subsection.

We do not intent to model the radiation pattern of any lamp in the building; apart that it is quite difficult to do it precisely, we do not see it too practical since there are simpler methods to detect light spots, which is our main goal. To get an idea of the complexity of any model, we first have to take into account that the maximum light illuminance that is expected under a light, is ideally modeled by the inverse square distance law, however that basic law is modified by the typical reflectors in common lamps, as well by the lamp diffusers. Other near-by reflectors, such as walls or furniture (mirrors) can influence as well in the perceived illumination from a lamp. Moreover, the output of the illumination sensor depends also on the angle, *ϕ*, of the sensor's orientation with respect to the lamp-to-user axis (see Figure 8); the Lambert's law states in that case that the illuminance is *E* = *I* · cos(*ϕ*)/*R*^2^. This angle *ϕ* depends not only on the tilt of the sensor but also on the relative position between the sensor and the lamp.

Obviously, the Light-matching approach can only work when the lamps are switched-on and the light sensor has a Line-of-Sight (LOS) with the lamp (e.g., if using a smartphone as in in Figure 1). The effect of having some switched-off lights in the building only has the inconvenient of getting less detections, so the pruning of location hypotheses proceeds more slowly.

#### 3.2.2. Light Spot Detection

We use the change in the registered illuminance to detect a light spot. When the user approaches a switched-on lamp the illuminance captured by the light sensor grows, then reaches a maximum when the user gets closest to the lamp, and finally decreases as he gets further from the light spot. The ideal peak-like illumination pattern occurs if the user passes exactly below a lamp. However, in order to detect as many light spot as possible, we also want detections when the user passes close to the lamp (typically about 1 m). The shape and height of the peak must be processed in order to robustly detect light spots.

The algorithm used for light spot detection consists of the following five stages:

- *Low-Pass Filtering.* We apply a low pass filter to smooth the illuminance sensor values. The smoothing is made with a 4th-order Butterworth Infinity-Impulse-Response (IIR) filter with a cut-off frequency of 1 Hz.
  (8) $$E_{f}(t)=\sum_{p=0}^{3}E(t-p)*b(p+1)-\sum_{p=1}^{4}E_{f}(t-p)*a(p)$$
  where *a* and *b* are the coefficients of the filter, and *E_f_* is the filtered illumination. This filter has a phase delay of 4 radians at 1 Hz (delay_filt_ = 4 rad).
- *Derivative of Illumination.* The derivative of the illumination keeps all the information of a peak and removes the irrelevant constant illumination levels of any particular room. We differentiate consecutive filter illumination values as follows:
  (9) $${\dot{E}}_{f}(t)=\{\begin{array}{ll} \frac{E_{f}(t)-E_{f}(t-1)}{T_{s}} & if\;E_{f}(t)\leq 1000_{\text{lux}}\\ 0 & \text{otherwise} \end{array}$$
  where *T_s_* is the sampling interval. In order to reject any light transitions that could occur when transferring from outside to inside, and vice versa (enormous light changes), we flatten the derivative curve if the filtered illuminance *E_f_* is larger than a given threshold (values lower than 1,000 lux are typical indoors).
- *Binarization of the Derivative.* We apply a simple thresholding in order to extract the sections where there is a steady illumination grow, and the complementary sections that contain a systematic illumination decrease. The threshold value used to select the relevant changes in illumination is 200 lux/s:
  (10) $${\dot{E}}_{f}^{\text{Binary}}(t)=\{\begin{array}{ll} 1 & if\;{\dot{E}}_{f}(t)\geq 200_{\text{lux}/\text{s}}\\ -1 & if\;{\dot{E}}_{f}(t)\leq -200_{\text{lux}/\text{s}}\\ 0 & \text{Otherwise} \end{array}$$
  The selected threshold can be obtained analyzing the rate of change of the illumination *E* with respect to the change of the distance *R* to a lamp installed at a given height *h*, as: *Ė* = *dE*/*dt* = *dE*/*dx* · *dx*/*dt* = −2 · *x* · *v* · *E*_0_/(*x*^2^ + *h*^2^)^2^ where *x* is the distance to the lamp projected onto a horizontal plane, and *v* = *dx*/*dt* is the displacement speed of the person. *E*_0_ = 5, 000 lux is a reference illumination term at the shortest distance (*x* = 0 or *R* = *h*), which also takes into account an average concentration factor of typical lamp reflectors. For a height of *h* = 2.3 m, the maximum illumination rate *Ė*_max_ is obtained at a distance to the lamp of *x* = 1.2 m.
- *Peak detection.* A peak corresponds to a zero-crossing in the derivative of the filtered illumination, if it is surrounded at both sides by significant positive and negative illumination gradients. A way to robustly detect this zero-crossing is to check the fulfillment of the next conditions:
  (11) $$\text{LD}(t)=\{\begin{array}{ll} 1 & \text{if}{\dot{E}}_{f}^{\text{Binary}}(t)=-1\&\\ & {\dot{E}}_{f}^{\text{Binary}}(t-\Delta t)=0\&\\ & \sum_{i=2}^{w}{\dot{E}}_{f}^{\text{Binary}}(t-i\Delta t)>1\&\\ & {\text{SET}}_{i=2}^{w}\{{\dot{E}}_{f}^{\text{Binary}}(t-i\Delta t)<0\}=\emptyset \\ 0 & \text{Otherwise} \end{array}$$
  that is to say, a light detection event (LD = 1) is generated if there is a transition from 0 to −1 in the binarized value (stated in the first two logical conditions;, and if the past samples (in a window of size *w*) there is at least 1 binarized value (third condition), and none of the samples in that window has a 0 value (last condition). This final checks are needed to robustify the detector, avoiding potential consecutive detections in a row which are unrealistic when passing under a single light.
- *Delay compensation.* Since the time *t*, at which a light spot is detected LD(*t*), is delayed by the filter and by the binarized zero-crossing, the time *t*_measu_(*k*) at which the user should have detected the lamp is computed as: *t*_measu_(*k*) = *t* − *t*_filter_ − *t*_ZC_. This corrected time of measurement is used to weight the particles in the PF, by interpolating the particles' states between consecutive step detections using Equation (5). The delayed LD causes a measurement update delay in the PF reweight process, but it does not cause any delay in the filter's position estimation which is performed in real-time.

**Figure 9.**
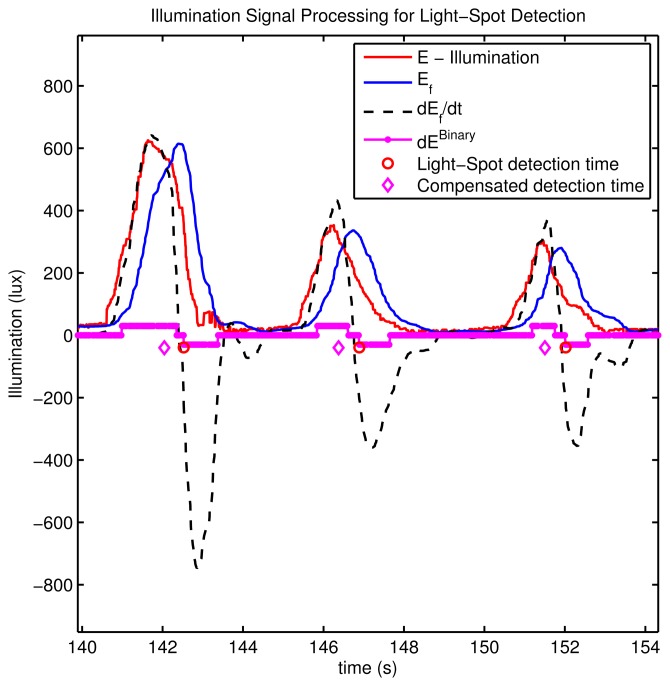
Processing of the illumination data *E* to detect light spots. Real example for a person walking at constant speed in a corridor holding a smartphone (Samsung Galaxy S-3) on his hand.

In Figure 9 this light spot detection process is shown for some real tests in a building with some fluorescent lamps. In this figure we show the original illumination as captured by the sensor *E*, all the intermediate processed signals (*E_f_*, *Ė_f_*, ${\dot{E}}_{f}^{\text{Binary}}(t)$), as well as the time detections.

It is important to remark that we are assuming that the person holds steadily the sensing device while walking under a lamp. If the person manipulates the device and changes the orientation with respect to the lamp, then some unexpected peaks in the illumination pattern could be registered. This could cause some false light detections that might deteriorate the location estimation. However, the inclination of the light sensor could be easily estimated, for example from the Attitude and Heading Reference Systems (AHRS) integrated in current smartphones or tablets. In this way we could compensate any illumination changes caused by inclinations of the sensor, or at least to check that the inclination was limited during the peak detection stage before validating a light spot detection.

One important topic is the influence of the solar light intensity that can enter in a building across windows. In principle this information can give clues about the presence of the user in a room that is at the perimetry of the building. In our current implementation, as this data has a totally different nature, we do not use it at all, in fact we implemented a method to detect illumination peaks that are caused by windows in order to reject its usage in the Light-matching approach. Alternatively, the light-matching concept could be extended to “natural areas of light”, areas which are illuminated by outside light.

#### 3.2.3. Lamp Database

The lamp database is just a list with the features of each lamp in a building. We annotate its 2D position (*r_l_*), the height with respect to the floor (*H_l_*), the size of the illumination section in terms of its length (*L_l_*) and width (*W_l_*) (*L_l_* = *W_l_* for circular/square lamps), and the orientation (*θ_l_*) of the larger axis of symmetry with respect to the North, *i.e.*, the lamp database contains {*r_l_*, *H_l_*, *L_l_*, *W_l_*, *θ_l_*} for *l* = 1… N. In our current implementation, we do not include any other feature related to the intensity flux of the lamp, as the power, radiant flux lobe, *etc*.

#### 3.2.4. Light Measurement Model

**Figure 10.**
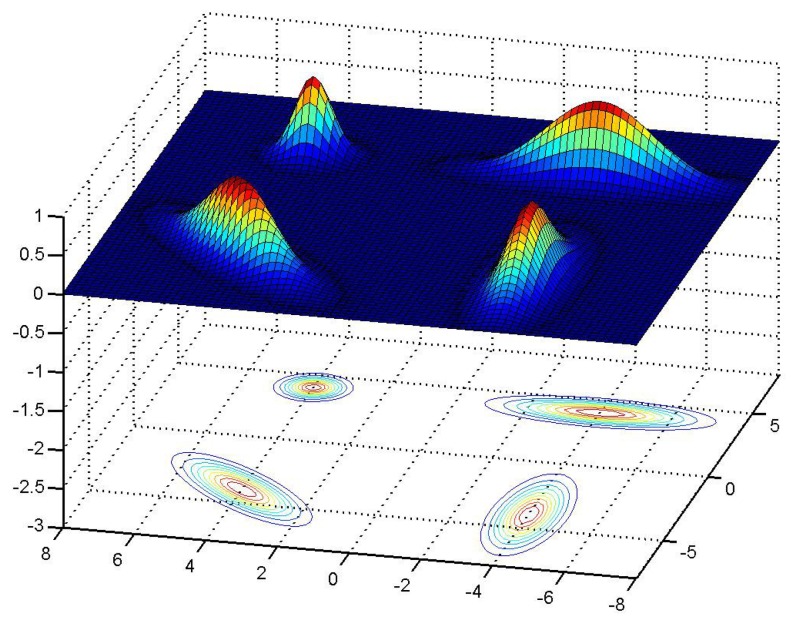
Example of the probability density function *P_l_*(LD(*t*_measu_[*k*])|*r̂*^(^*^i^*^)^[*k*]) for 4 lamps. Lamps are located at positions {(−4, −4), (4, −4), (−4, 4), (4, 4)}, three of them are fluorescent lamps of length *L_l_* = 1.2 m and width *W_l_* = 0.2 m, which are oriented at angles 0, 45 and 90 degrees. The other lamp at position (4, 4) is a small size symmetrical bulb light.

This Light-based measurement model is ambiguous by nature; *i.e.*, when we detect a light spot we know that the user is under a switched-on lamp, but we do not know which one since they are not codified by any method. Consequently the measurement model is multi modal, *i.e.*, is formed by a mixture of probability distributions centered in the lamp's position. With this measurement model the weight update of each particle in the PF is done as follows:

(12) $$w^{\left(i\right)}[k]=w^{\left(i\right)}[k-1]\cdot P(\text{LD}(t_{\text{meas}}[k]){|\hat{r}}^{\left(i\right)}[k])$$

where *P*(LD(*t*_measu_[*k*])|*r̂*^(^*^i^*^)^[*k*]) is the probability of getting a light detection (LD(*t*_measu_[*k*] = 1) when the user is at position *r̂*^(^*^i^*^)^[*k*]. This multimodal probability is modeled as the sum of all the probability distributions of each Lamp in a building floor:

(13) $$P(\text{LD}(t_{\text{meas}}[k])|{\hat{r}}^{\left(i\right)}[k])=\sum_{l=1}^{L}P_{l}(\text{LD}(t_{\text{meas}}[k])|{\hat{r}}^{\left(i\right)}[k])$$

where *L* is the total number of lights in a floor, *l* is the index of a particular lamp. We propose to use a standard two-dimensional normal distribution centered at the lamp's position, and with a covariance matrix adapted to the size and orientation of the lamp, in order to model the probability of detection of each lamp:

(14) $$P(\text{LD}(t_{\text{measu}}[k])|{\hat{r}}^{\left(i\right)}[k])=\frac{1}{2\pi \sqrt{\left|\Omega_{l}\right|}}\exp\{-0.5({\hat{r}}^{\left(i\right)}\left[\text{k}\right]-r_{l})\Omega_{l}^{-1}{\left({\hat{r}}^{\left(i\right)}\left[\text{k}\right]-r_{l}\right)}^{T}\}$$

where *r_l_* is the position of the *l* lamp, and Ω*_l_* is a covariance matrix that defines the area around the position of lamp *l* where it is probable to detect it. We create matrix Ω*_l_* from the eigenvalues and eigenvectors that define an ellipsoidal distribution of length *L_l_*, width *W_l_*, and with its main axis oriented an angle *θ_l_* as:

(15) $$\Omega_{l}=\lambda [1]\cdot v_{\text{eigen}}[1]v_{\text{eigen}}{\left[1\right]}^{T}+\lambda [2]\cdot v_{\text{eigen}}[2]v_{\text{eigen}}{\left[2\right]}^{T}$$

where the eigenvalues are λ[1] = (*L_l_* + 0.3)^2^, λ[2] = (*W_l_* + 0.3)^2^ and the eigenvectors are: *ν*_eigen_[1] = [cos(*θ_l_*), sin(*θ_l_*)]*^T^* and *ν*_eigen_[2] = [cos(*θ_l_* + *π*/2), sin(*θ_l_* + *π*/2)]*^T^*. Figure 10 shows an example of the aggregated probability density functions of 4 lamps.

### 3.3. Additional Measurements Models

In the general localization framework presented in Figure 6 any additional measurement can be integrated using this general weight update:

(16) $$w^{\left(i\right)}[k]=w^{\left(i\right)}[k-1]\cdot P(z[k])|{\hat{X}}^{\left(i\right)}[k])$$

which is adapted for each particular type of measurement:

- *Magnetometer.* If a magnetometer provides the estimation of the heading of the user *θ*_magne_ then we can update particle's weight as:
  (17) $$P(\theta [k]|{\hat{X}}^{\left(i\right)}[k])=\frac{1}{\sqrt{2\pi }\sigma_{m}}\exp\{-\frac{{\left|\Delta \theta^{\left(i\right)}\right|}^{2}}{2\sigma_{m}^{2}}\}$$
  where *Δθ*^(^*^i^*^)^ = *θ*_magne_ − *X*(4)^(^*^i^*^)^ and *σ_m_* is the uncertainty of the electronic compass's heading estimation (*σ_m_* = 0.68 rad).
- *RSS (WiFi/RFID/Bluetooth).* Assuming that we measure the signal strength RSS[*k*] to an WiFi access point, or any other RF source such as RFID or Bluetooth tags, we can update the weights of each particle as:
  (18) $$P(\text{RSS}[k])|{\hat{r}}^{\left(i\right)}[k])=\frac{1}{\sqrt{2\pi }\sigma_{\text{RSS}}}\exp\{-\frac{{\left|\Delta {\text{RSS}}^{\left(i\right)}\right|}^{2}}{2\sigma_{\text{RSS}}^{2}}\}$$
  where ΔRSS^(^*^i^*^)^ = RSS[*k*] − (RSS_0_ − 10*β* log_10_(‖*r̂*^(^*^i^*^)^[*k*] − *r*_ap_‖)) being *β* the path loss exponent, RSS_0_ the expected signal strength at a reference distance of 1 m, and *r*_ap_ the position of the WiFi access point, RFID or Bluetooth tag, *etc*. A typical value for the standard deviation of these RF sources is about 6 dB (σ_RSS_ = 6 dB).
- *Map-matching*. The weight of a particle is set to zero,
  (19) $$P(r^{\left(i\right)}[j]|r^{\left(i\right)}[j],r^{\left(i\right)}[j-1])=0$$
  whenever the segment connecting two consecutive step locations (*r*^(^*^i^*^)^[*j*], *r*^(^*^i^*^)^[*j* − 1]; intersect any wall of a building floor map.

This section has detailed our particular Light-matching implementation, including the extension for using other sources of information available from unmodified buildings. Next sections evaluate the performance of the Light-matching concept and the benefits of integrating it with complementary signals of opportunity.

## 4. Evaluation: Simulated Results

In this section, we evaluate the convergence of the location hypothesis while a person is moving in a simulated indoor space. Additionally, we evaluate the location accuracy that can be obtained for different fusion combinations using PDR, Light-Matching, Magnetometer, WiFi, RFID or Map-matching. In these tests we use the particle filter approach presented in last Section 3, with 10,000 particles. The measurement models used to generate the synthetic data (WiFi and Magnetometer) are the same as presented in Subsection 3.3.

### 4.1. Convergence of Location Hypothesis

In Section 2 we analyzed the dependance of the number of location hypotheses with the number of detected lights. We obtained an algebraic expression (Equation (1)) that was valid for the case of aligned regular-distributed lamps and for a person walking along a straight path. The generalization of this expression in the 2D case was not possible since it depends on many variables, such as the particular lamp distribution and the selected trajectory of the user, both of which change the number of light detections, and, consequently, the number of location hypotheses. In this section, we perform several simulations to get an idea of the dependance of the number of hypotheses with the number of light detections in a 2D case, as well as, how the speed of convergence to a single location hypothesis is influenced by the regularity of the lamp distribution, the use of the magnetometer, or other information such as map-matching.

**Figure 11.**
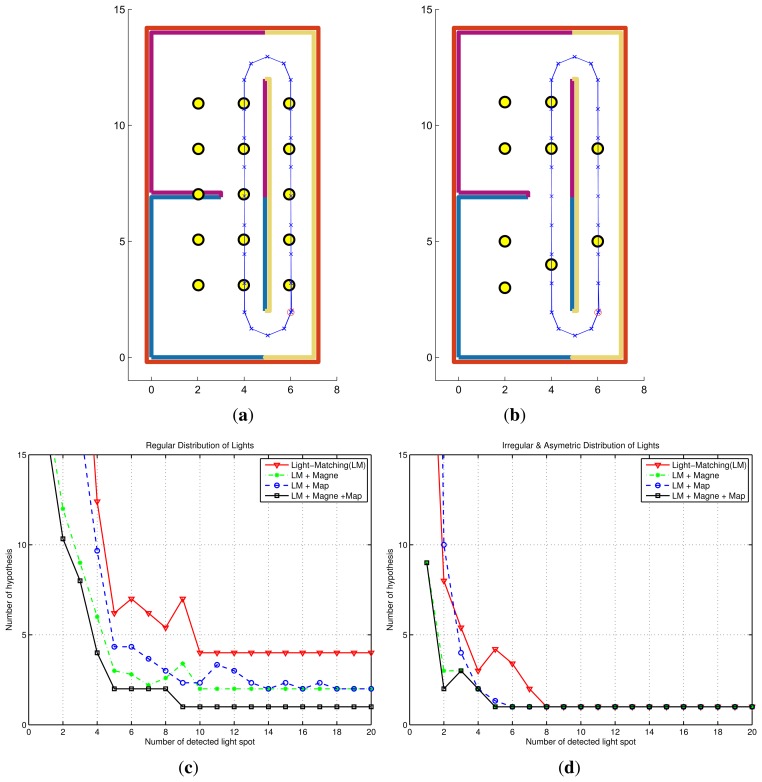
Simulated 2D analysis of the dependance of the number of location hypotheses with the number of detected lights: (**a**) environment with regular light distribution; (**b**) environment with an irregular and asymmetrical light distribution; (**c**) Evolution of the number of hypotheses for the regular case; (**d**) Evolution of the number of hypotheses for the irregular case.

In Figure 11a and b we can see the simulated environment, which has an area of 98 square meters (14 by 7 m), and a wall distribution that defines a vertically-aligned corridor at the right, and two rooms at the left. There are two different lamp distributions: in Figure 11a 15 lights are distributed regularly with an inter-lamp distance of 2 m, whereas in Figure 11b the lights are distributed in an irregular way, with different gaps between lamps: 2 and 4 m, as well as some asymmetries, as the one generated by the three lights at the lower-left room. The simulated walking trajectory is also overlaid on the map in blue color; the crosses represent the stances and the small circle the initial and final position of the path. The trajectory passes under some of the lights in the environment but other lamps are not visited, as in a real case. The trajectories are long enough to obtain 20 light detections in each case (*i.e.*, 2 cycles for the regular case and 4 cycles in the irregular case).

The evolution of the number of hypotheses for the regular light distribution is shown in Figure11c. The number of clusters in the arbitrary distribution of particles is calculated automatically using a hierarchical binary tree clustering algorithm, which measures the distance among particles in the four-dimensional space { $r_{x}^{\left(i\right)}$, $r_{y}^{\left(i\right)}$, cos(*θ*^(^*^i^*^)^), sin(*θ*^(^*^i^*^)^)}. Four localization algorithms are compared: (1) the basic *Light-matching* (LM) algorithm (which includes the PDR subsystem); (2) the Light-matching approach augmented with information from the magnetometer; (3) the Light-matching approach augmented by map-matching; and (4) the Light-matching approach augmented with both magnetometer and map-matching. We can see in Figure 11c that the initial number of hypotheses is high (larger than the number of lamps), as expected, but that it decreases after some additional detections. In the standalone LM approach, there is no convergence, and a minimum of 4 hypotheses persist if no more information is used. This number reduces to two potential locations if the magnetometer or the map are used. However the use of all the available information makes the location algorithm converge to a single hypothesis after the ninth light detection.

In the case of an asymmetrical light distribution, as already expected from the analysis in Section 2, the convergence is improved significantly (see Figure 11d). Even with the standalone LM method, it is possible to reach convergence after the eighth detection. Using some additional information, a single hypothesis is obtained after the fifth detection.

These simulations, which are just examples of the infinite possible configurations, give an approximate idea of how location convergence is influenced by the geometry of light distributions and by the use of additional information. In the real world, we know that we will benefit from irregular light distributions when changing from one room to another, and also from the use of as many signals of opportunity as possible.

### 4.2. Location Accuracy

In this section we will evaluate the positioning accuracy of the proposed method through simulated tests with synthetic positioning data. We will offer results using the Cumulative Density Function (CDF) of the 2D positioning error with respect to the ground-truth trajectories. The CDF measures the probability that our positioning system has an error less than a given value.

**Figure 12.**
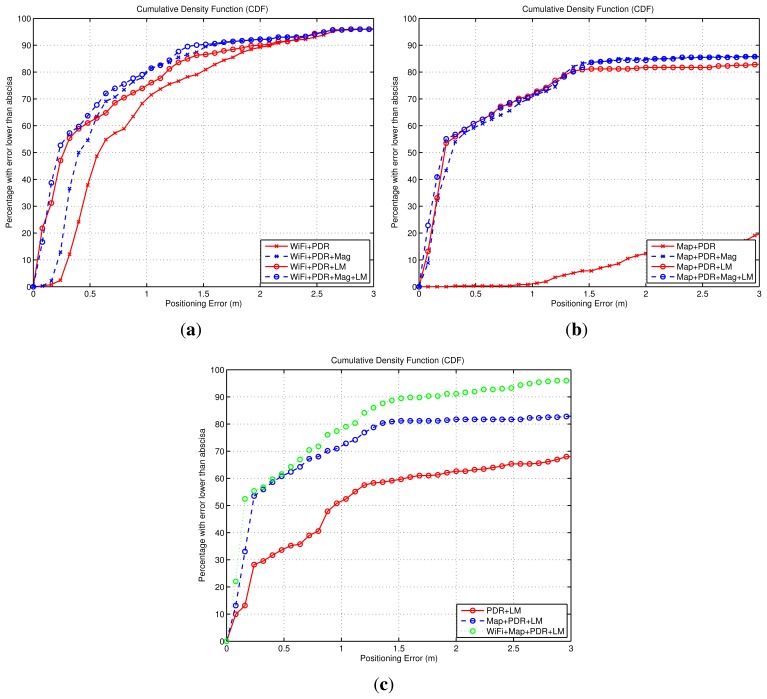
Cumulative Density Functions (CDF) of the positioning error, obtained from simulated trajectories corresponding to the scenario of Figure 11b. The three CDFs correspond to: (**a**) Benefit obtained in WiFi-based positioning techniques by adding the Light-Matching (LM) method, as well as a magnetometer; (**b**) Benefit obtained in Map-maching-based positioning techniques by adding the Light-Matching (LM) method, as well as a magnetometer; (**c**) Positioning errors with the Light-maching method alone, and the benefits obtained by LM when other methods are fused (Map-matching and WiFi positioning).

The objective of this discussion is to compare the improvements that can be achieved with different fusion combinations. First, we evaluate how a WiFi-based positioning solution assisted with relative Dead-Reckoning information is improved by using the Light-matching (LM) method or the magnetic information. In Figure 12a we can see that the basic WiFi + PDR fusion approach has an error of 1 m or lower for 70% of the cases. When the magnetic information is used (WiFi + PDR + Mag) the accuracy is improved significantly (≤0.7 m at 70%). When the Light-matching method is used (WiFi + PDR + LM), an important reduction of the errors is observed, specially at the lowest range of errors. The improvement is similar when using all the measurements (WiFi + PDR + LM + Mag). The LM improvement (traces with circle markers in Figure 12a *vs.* those with cross markers) is due to position corrections happening during light detections that concentrate the particles around a lamp position, resetting any accumulated drift error. Note that a significant part of the trajectories (about 20%) has an error quite larger than 1 m, arising from the initial sparse distribution of particles around the simulated indoor area, since no initial position nor orientation is given. When enough information is received (from WiFi or Light-detections) the initially spread, or even multimodal, distributions start to concentrate and the accuracy gets better than 0.3 m in most of the cases. From this discussion, the benefit of using Light-matching (LM) information to complement WiFi or RSS based positioning is clear.

We also analyzed the benefit of the LM concept, when used in parallel to a Map-matching location method assisted by relative Dead-Reckoning information. In Figure 12b it can be seen that for this particular simulation, the basic Map-matching with inertial information (Map + PDR) has a poor performance. This is caused by a multimodal particle distribution, in which two location hypotheses are formed: the right one, and a symmetric and wrong hypothesis. The weighted average of the particles (Equation (7)) gives a position estimation close to the center of the indoor area. When we add the magnetic information (Map + PDR + Mag) this ambiguity is eliminated and the location accuracy is improved (lower than 0.9 m in 70% of the cases). The LM approach also eliminates the location ambiguity, and the results are similarly good in both cases (Map + PDR + LM or Map + PDR + LM + Mag). As a conclusion, in map-matching approaches to localization, Light-matching can be used additionally (on its own, or together with magnetometer or WiFi positioning information) in order to reduce location ambiguities and achieve convergence to a unimodal probability density function.

We finally studied how the basic version of the LM approach (PDR + LM) can be improved by the addition of other sources of information. Assuming that LM is possible at all (smartphone held in hand, sufficient lights turned on, *etc.*), the main weakness of the PDR + LM approach is the delay until convergence, as was explained in Section 4.1. Obviously, this problem usually appears in the initial part of the position estimate, and would normally disappear as the trajectory gets longer. The error caused by convergence is shown in the CDF curve of Figure 12c, where the positioning error of the PDR + LM method is, for half of the estimations, larger than 1 m. With the assistance of Map-matching (Map + PDR + LM), or an absolute location method such as WiFi-based positioning (WiFi + Map + PDR + LM), delays to convergence are reduce, and the accuracy gets better than 1 m for 70% of the cases. As a conclusion, the combination of matching approaches (Light- or Map-based) with absolute positioning techniques is always a good fusion scheme if available, since they eliminate multi-hypotheses and permit to reach sub-meter positioning accuracy.

The above results use a fixed dead-reckoning performance, with ideal, noise-free IMU signals. In order to complement this study, we have also evaluated how the combined light and inertial estimation (PDR + LM) performs for different uncertainty settings. It is known that the noise in the IMU signals causes an error in the PDR estimated distance and orientation [38–41]. The quality if the PDR estimation is expected to affect directly the performance of the light-matching approach, because a larger uncertainty in the PDR estimate will need a larger spread of the particles around the true position, and consequently a lightspot detection can cause the reinforcement of additional wrong hypotheses that correspond to those particles compatible with a wrong nearby lamp.

The performance of a PDR estimation is mainly determined by the quality of the gyroscope used. This is normally specified by measuring their bias Instability (BRW or Bias Random Walk), and by their white noise content, which is measured with an Angular Random Walk (ARW) estimate. Both parameters (BRW and ARW) can be determined by studying the Root Allan Variance of long duration gyroscope signals [39]. Typical MEMS-type IMU sensors usually have an ARW in the range of 0.25–5 ^°^/ $\sqrt{h}$ (degrees/square root of an hour) and a BRW between 10 and 60 ^°^/*h*. With these figures into consideration, we have added different noise content to the ideal IMU signals and repeated the simulations several times for each case. The median localization error for each case is summarized in Table 2. It can be seen that the performance is close to the ideal case when the IMU error is below 5 ^°^/ $\sqrt{h}$ and 30 ^°^/*h* (cases 1 and 2 in Table 2), *i.e.*, using recent MEMS IMU technology. With lower performance IMUs (cases 3 to 5 in Table 2), the PDR + LM approach starts to deteriorate progressively as expected.

## 5. Evaluation: Experimental Results

**Figure 13.**
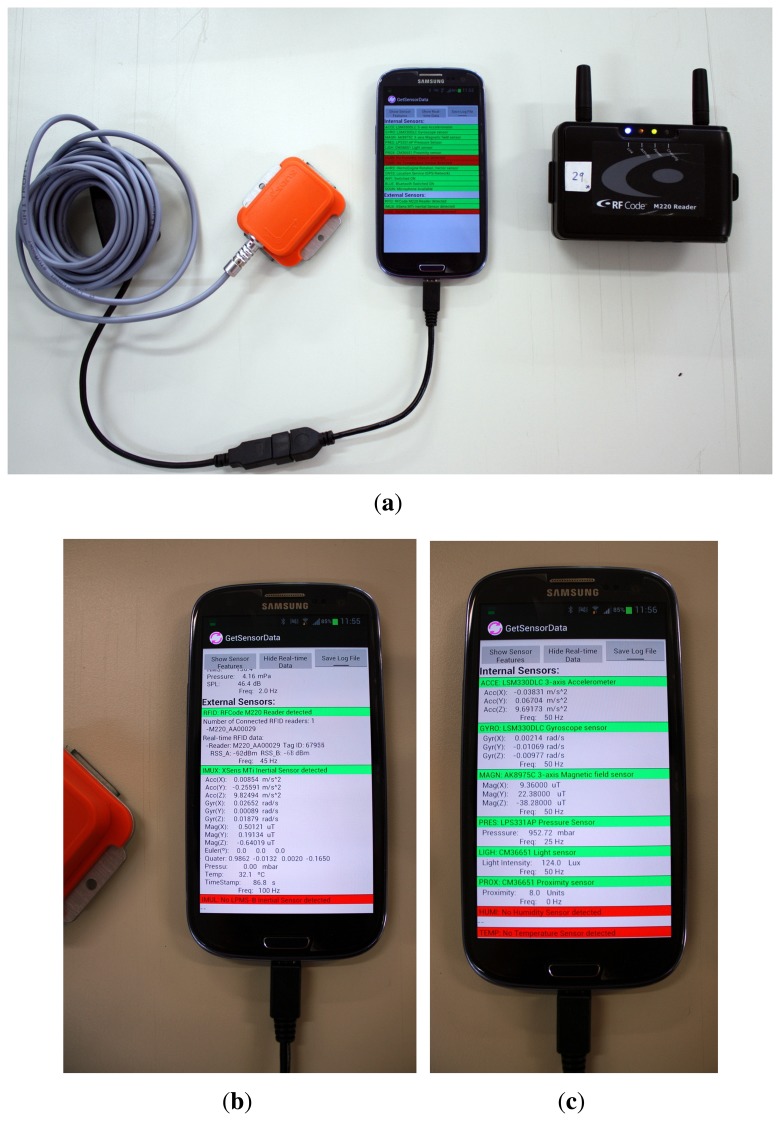
Equipment that was used by a person to capture all the sensor data. (a) Samsung Galaxy S3 mobile phone connected to an Xsens Mti inertial unit (USB connection) and to an RFID RFCode reader (Bluetooth connection). The developed “GetSensorData” Android application runs in the S3 phone and collects data from both external (Xsens and RFcode) and internal sensors; (**b**) A detail of the information captured from the external sensors; (**c**) A detail of the information captured from the phone's internal sensors (among others the Illumination, GNSS, WiFi, Sound, *etc.*).

We have empirically tested the Light-matching technique in walking trajectories in our CAR-CSIC building. The user under test (Figure 1) carries with him (Figure 13) a foot-mounted IMU (Xsens model MTi), an RFID reader (model 220 from RFCode), and a Samsung Galaxy S3 mobile phone. An Android application running on the smartphone collects data from the external inertial sensor and the RFID reader, as well as readings from several internal sensors (the GNSS estimated position, the WiFi Signal Strength and the Illumination, among others). A log file with the received sensor information is then processed off-line in a desktop computer using a Matlab software implementation of the estimation methods described in previous sections.

**Figure 14.**
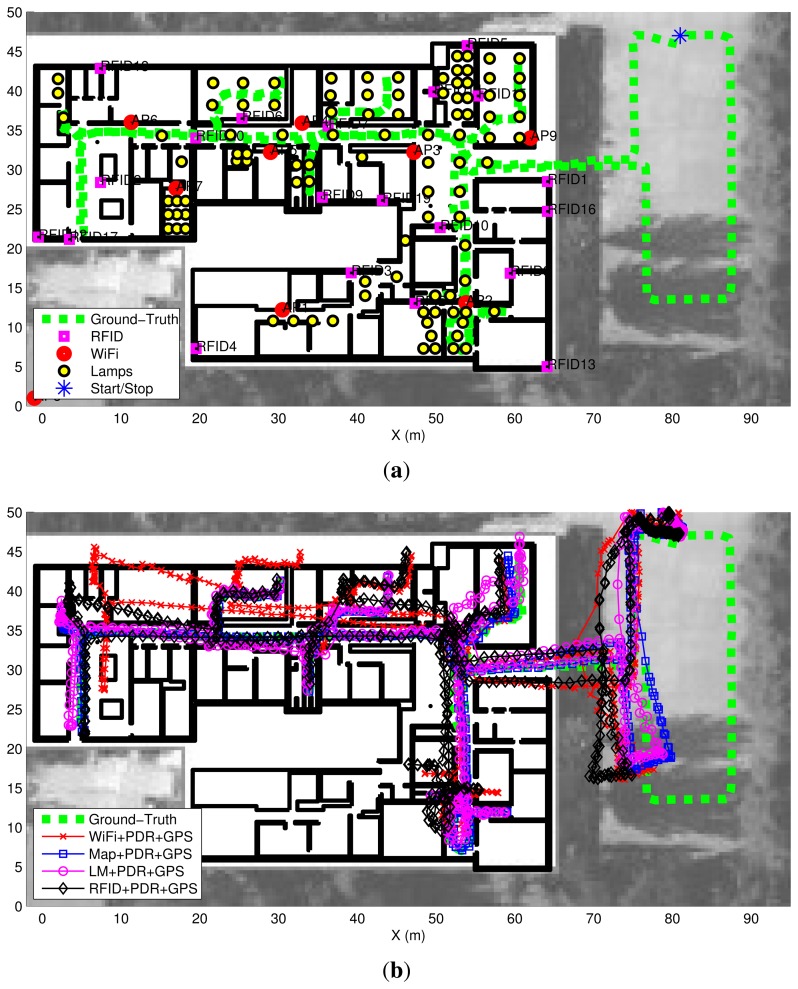
Experiments at CAR-CSIC building. (**a**) Ground-truth trajectory thats starts and ends at the same point (outside the building). Light-Lamps are marked as well as the Wifi access points and RFID tags; (**b**) Estimated trajectories for some of the different fusion tests.

Figure 14a shows (dashed green line) the experimental trajectory, with the start and end at the same point outside the building (blue asterisk), and total duration of 10 min. Additionally the system requires:

- A map of the building,
- Coordinates and characteristics of the lamps,
- RFID and WiFi access points' locations.

This information is represented in Figure 14a as explained in the figure's legend.

In contrast, to the simulated case, where we had a real ground-truth, in this experimental tests we only have an approximate ground-truth, which is the one we will use to estimate the localization errors. This reference path is created by using all the measurements available (including the Map-matching approach, LM, GPS, RFID and WiFi) and additionally giving the true initial position and orientation to the initial state of the particle filter. By doing it we obtain a reliable path estimation outdoors (where GPS is not precise enough, but can be improved with PDR's movement model) and also indoors (where Map-matching performs very well in this highly partitioned building).

The lamp database was created by visiting several rooms of our building and measuring the coordinates of the lamp with respect to the room's walls with a TOF-laser-based distance meter (accuracy of 0.1 m). The lamp position, orientation and size are annotated on the calibrated floor map. Not all the lamps in the building were stored, and from those, not all were switched on during the tests. There was a great variation in lamp light intensity, size and orientation, as well as lamp density per area in our building.

With respect the RFID tags, which were fixed at some walls in the building, only 20 tags were showed in Figure 14a, because this is the number of tags used for most of the location tests, but a total of 100 tags were installed and registered by the system. The RFID measurements were processed using the signal strength (SS) attenuation model presented in Section 3.3 with a path-loss exponent equal to 2.3, as already used by the authors in previous works of RFID localization [11].

It is important to mention that in the experimental tests, in contrast to the simulated tests, the GNSS position information, provided by the S3 phone, is used. The GNSS information is a fusion of the American GPS and the Russian Glonass systems. More than 14 satellites are typically visible and used outdoors. Inside this one-story building several satellites signals (between 6 and 9) are still received, but due to indoor multipath the quality of positioning is much lower than outdoors (error of about 30 m in most indoor positions) and therefore the data from GNSS, although used in the fusion process, is less useful.

The result of the position estimation using different fusion combinations are presented as trajectories on top of a location map in Figure 14b. Four different algorithms are represented:

- Wifi + PDR + GPS
- Map + PDR + GPS
- LM + PDR + GPS
- RFID + PDR + GPS

The first significant aspect that we observe is that the outdoors estimation of the GNSS system is quite degraded, with an error of more than 10 m (difference between the dashed-green ground-truth path and the other estimated paths). The black and red traces (Wifi + PDR + GPS and RFID + PDR + GPS) are slightly biased at some areas of the indoor space (the black around the point [50, 10] m, and the red around the point [10, 40] m). The other two estimations (Map + PDR + GPS and LM + PDR + GPS) are significantly better than the RSS-based estimations, the Map-matching approach being the best.

**Figure 15.**
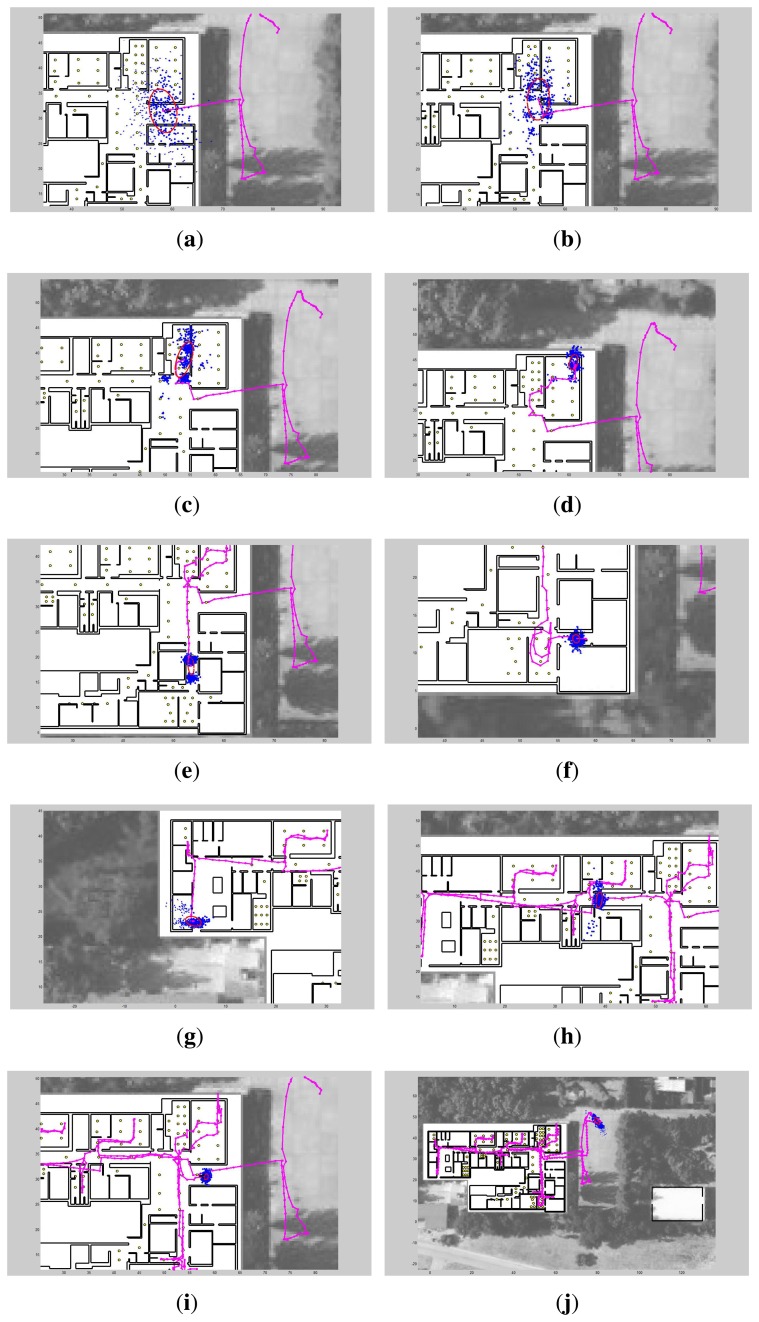
Some intermediate snapshots of the Light-matching estimation experiments at CAR-CSIC building. The GNSS is used outdoors, and PDR with Light-matching is used to improve the accuracy of the position estimation indoors.

As we are specially interested in showing the performance and limitations of the LM subsystem, we present several snapshots of the intermediate LM + PDR + GPS algorithms. Figure 15 shows 10 snapshots in chronological order, containing the cloud of particles, an ellipsoid representing the estimated position covariance, and the partial estimated trajectory. In Figure 15a, just before entering the building, the cloud of particles is quite disperse, representing the uncertainty in the GNSS estimation, which is the one that predominates. In Figure 15b after the first light detection, the cloud of particles is transformed into a set of near-by Gaussian hypothesis with a distribution that reflects the arrangement of lamps installed in the hall of the building. This multimodal hypotheses are shaped in the next lamp detections, as presented in Figure 15c–e. Regarding the two hypotheses shown in Figure 15e, the lower one is the correct. The first unimodal distribution is observed in Figure 15f. After that the trajectory remains unimodal and fitted closely to the ground-truth, except for a period with no light detections in the corridor, that finally is corrected in Figure 15h after one detection. Finally the complete trajectory with the final particle distribution is shown in Figure 15j which is the same case as the LM + PDR + GPS result presented in Figure 14b.

**Figure 16.**
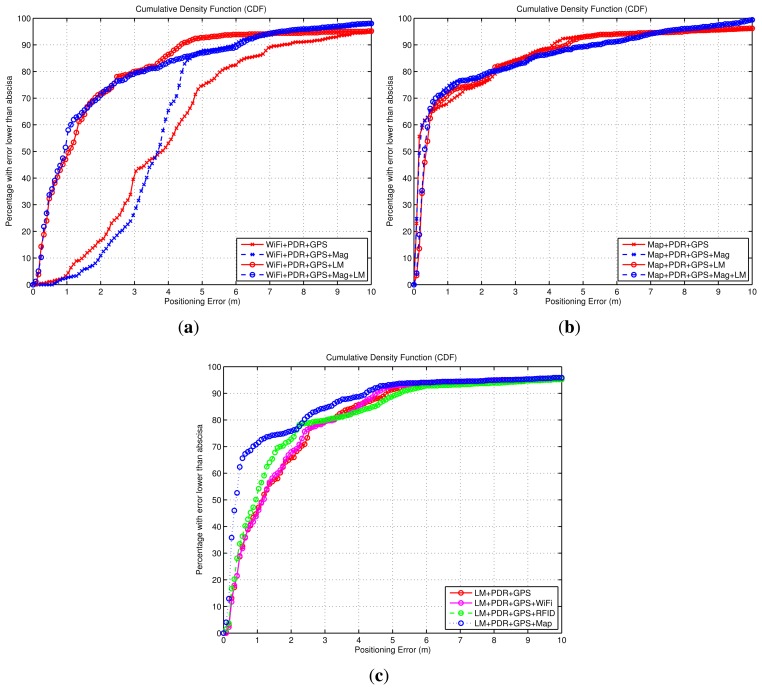
Positioning accuracy by means of Cumulative Density Functions (CDFs). The data is experimental for a test with a ground-truth trajectory as in Figure 14a. The three CDFs correspond to: (**a**) Benefit obtained in WiFi-based positioning techniques by adding the Light-Matching (LM) method, as well as a magnetometer; (**b**) Benefit obtained in Map-matching-based positioning techniques by adding the Light-Matching (LM) method, as well as a magnetometer; (**c**) Positioning errors with the Light-matching method alone, and the benefits obtained by LM when other methods are fused (Map-matching, WiFi and RFID positioning).

Apart from the analysis of the four algorithms presented in Figure 14b and Figure 15, additional types of fusion combinations were analyzed. Their performance is shown in Figure 16 using a CDF representation, and the same criteria as in the simulation analysis of last subsection.

The experimental CDFs shown in Figure 16 show that the WiFi-based positioning is improved significantly when we add the LM approach (Figure 16a), and that the Map-matching approach works very well when no ambiguities appear in the building floormap, and that the LM method can not add any benefit in that case (Figure 16b). That is similar results to the conclusions obtained from the simulated data in Figure 12. When we try to improve the basic LM method (LM + PDR + GPS) adding WiFi, RFID or Map-matching information, we obtain the CDFs in Figure 16c. The Wifi contribution is not perceptible, the RFID data slightly improves the estimation, and the addition of map-matching provides a good benefit; this improvement is mainly due to the elimination of the initial multiple hypotheses generated by LM. Note than in all cases about 15% of the samples are contaminated with errors larger than 4 m; this error is due to the GNSS estimation that is equal for all methods outdoors. As a conclusion, the best solution is the one that combines more sources of information, as in the case (Map + PDR + GPS + Mag + LM) in Figure 16b already presented, with an error lower than 1 m in 70% of the cases.

**Figure 17.**
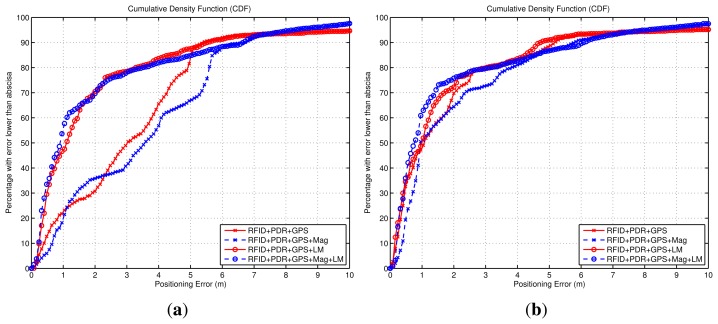
Cumulative Density Functions (CDFs) of positioning error in several circumstances, corresponding to the test trajectory of Figure 14a. The two CDFs correspond to: (**a**) Benefit obtained in RFID-based positioning techniques (using 20 RFID tags) by adding the Light-Matching (LM) method, as well as a magnetometer; (**b**) The same case as before, but using a total of 100 RFID tags in the building.

The benefit of the LM method is specially remarkable when acting as a complement to RSS-based positioning (e.g., WiFi as seen in Figure 16a). This improvement can be contrasted with another RSS-based positioning system such as RFID; in Figure 17 we evaluate it for the case of using 20 RFID tags and also using 100 tags, which is an over-populated tag deployment. For the normal, or low density of RFID tags (20 tags in 2500 m^2^), the accuracy improvement is from 4 m to 2 m (70% of the cases). In the case of a high tag density (100 tags), the improvement is lower but still significant (from 2.3 m to 1.3 m, in 70% of the cases).

## 6. Discussion

In this paper, we have presented the Light-matching (LM) idea, its implementation details and evaluation with simulated and experimental tests. It is important to mention that the LM approach is intended as a support to existing PDR solutions, refining estimates that otherwise would be affected by drift. We believe that an effective indoor positioning solution must use as much information as possible, so inertial information should be fused with other signals of opportunity such as the received signal strength from standard nodes (WiFi, RFID, Bluetooth, *etc.*), magnetic fingerprints, pressure information, map-matching, *etc.* or specific positioning beacons, such as ultrasound emitters, cameras, pseudolites, UWB ranging, and so on. Although the use of unmodified light indoors has already been addressed in some previous works, as cited in the introductory section, we explored a different approach that shows how this signal of opportunity can improve pedestrian dead-reckoning solutions. We defined a general-purpose multi-hypothesis localization framework where the ambient illumination can be effectively fused with other informative measurements in order to achieve accurate indoor positioning.

The illumination received from the lamps in a building, which is the signal of opportunity used by the LM concept, is not available in many situations, for example when the device does not have a light sensor, or if all the lights in the building are switched off, or when the phone is in the pocket, handbag, or protected by a cover. In these cases, the LM corrections will not take place, and localization must be performed using other sources of information available in the localization system. If the localization system is designed with only PDR and LM, and no light data is available, then the system will behave as a PDR alone system with significant drift after traveling a relatively long distance (typically with a positioning error of 1% to 5% of total traveled distance). However, once the user holds the phone at constant orientation in front of him (phone pointing upwards and with line of sight to ceiling lamps) to look at the phone's screen for his location or receive navigation instructions, LM can take place and improve the position estimation.

Assuming that the light data is available, the illumination gradient that is perceived when a person gets closer to a light spot can be used in different ways for localization, with different robustness results. The approach based on the illumination gradient for estimation of the range from the user to the light, can be used to estimate the traveled distance with decimeter accuracy in ideal conditions (a reliable model of light intensity), but has the drawback of being too sensitive to any illumination change (different diffusers among lights, different powers, convolution of close-by light, near windows, *etc.*). A different approach consist of using the illumination gradients under a light spot to detect a person passing under a light (light detections); it uses the dominant illumination intensity rise (when getting close to the light) followed by a decrease (getting apart from the light) to detect when a person has passed under, or close to, one light spot. This detection process is the one that was implemented and used in this paper and it is part of the LM concept. It has the advantage, by design, of being less sensitive to the disturbing effects noted above.

The statistical study of the light detection problem is an interesting topic. While misdetections (*i.e.*, the detection process misses a true light spot) are not harmful for the performance of our system, false alarms (detecting a light when there is none) can cause a significant deterioration of the location estimate. In this paper we have not included statistics of the light detection performance since the detection process worked quite ideally in our simulated and experimental tests. This ideal behavior of our light detector was achieved by designing a very conservative solution. The light detector was implemented using: (1) a low-pass filter to attenuate slight inclinations of the phone; (2) a derivative of illumination to observe the gradients; (3) a conservative binarization using a significant threshold value in order to detect only lightspot with a very clear up/down gradients; and (4) a robust looking backwards peak detection algorithm to avoid too consecutive detections. In this manner we get a moderate correct detection rate, but were able to avoid any potential false alarms for a looking-to-screen use of the phone (looking mode).

Undesirable light gradients can also be generated if the user rotates the smartphone significantly with a close-by source of light (e.g., when moving the phone to a pocket or holding the phone in hand while swinging the arm). Additionally, abrupt light gradients can be generated by switching on and off lights, for example when entering in rooms. These special and perturbing light gradients should be also detected and canceled out to make the light detection process robust enough in any situation. A method to increase the robustness of light detection can rely on exploiting the knowledge that we have on the orientation of the light sensor on the phone (its rotation matrix), the knowledge of the real displacement of the person (obtained by PDR) and the maximum size of lights in the database. Using this available information, it would be quite straightforward to define methods to reject all light detections generated while the phone is not kept in a static position (gradient generated from phone's rotations), while the user is motionless (it's impossible to pass under a light spot if the user is motionless), or if the gradient duration is larger than the largest lamp (then the gradient most likely comes from light from a window). These proposed approaches, as well as other more sophisticated should be defined and analyzed in order to evaluate the performance of the light detector under real phone use for long periods of time and in different buildings. We plan to perform this analysis in a future work, since the scope of this paper was to present the LM concept, the convergence problem and methods to prune hypothesis, present the implementations details, and to perform some tests under simulated and experimental conditions in order to assess the validity of the concept.

## 7. Conclusions

This paper has presented how inertial Pedestrian Dead-Reckoning (PDR) location systems can be improved with the use of a light sensor to measure the illumination gradients created when a person walks under ceil-mounted unmodified indoor lights. The process of updating the inertial PDR estimates with the information provided by light detections is a new concept that we denominated *Light-matching* (LM). The displacement and orientation change of a person obtained by inertial PDR was used by the LM method to accurately propagate the location hypothesis (movement model), and vice versa, the LM approach actually helps the PDR approach by providing an absolute localization and by reducing the typical PDR-alone drift. The LM approach, as any other matching technique, requires to know the 2D position, size and orientation of all lamps in a building, however the current lighting state (if they are switched on or off) is not needed.

We presented the basic description of the light-matching concept, and illustrated it with some examples. A key issue in LM is the generation and pruning of multiple location hypothesis. We showed that even from an initially unknown location and orientation, whenever the person passes below an switched-on light spot, the location likelihood is iteratively updated until the likelihood potentially converges to a unimodal probability density function. The time to converge to a unimodal position hypothesis depends on the number of lights detected and the asymmetries/irregularities of light distributions.

The LM concept is not designed to be used as an stand-alone solution for indoor location; it is just a complement that should be used whenever light data was available. Therefore, the LM approach should be used in cooperation with other signals of opportunity (WiFi, magnetic fields, pressure, map-matching, and so on) to obtain a high accuracy, robust and seamless indoor localization solution.

In this paper, after presenting the basic description of the light-matching concept, we provided the implementation details using a particle filter, and several simulated and experimental tests using a smartphone equipped with a light sensor. The performance of the integrated solution achieves a localization error that can be better than 1 m in most of the cases. Future interesting work should include the study of the robustness of the light detector, and the use of SLAM techniques to automatically obtain the lamp database.

## Figures and Tables

**Table 1.** Dependance of the number of location hypothesis with the number of lights present and the number of lights detected (case of lights aligned and a straight path).

**Number of Lights** (*n*_lights_)	**Number of Detections** (*n*_det_)	**Number of Hypotheses** (*n*_hyp_)
1	0	∞

	1	*^n^*quad

2	0	∞

	1	2·*n*_quad_

	2	2

3	0	∞

	1	3·*n*_quad_

	2	4

	3	2

4	0	∞

	1	4·*n*_quad_

	2	6

	3	4

	4	2

5	0	∞

	1	5·*n*_quad_

	2	8

	3	6

	4	4

	5	2

**Table 2.** Influence of the IMU noise content on the performance of the PDR + LM approach.

	Ideal case	Noise Case 1	Noise Case 2	Noise Case 3	Noise Case 4	Noise Case 5
ARW (^°^/ $\sqrt{h}$)	0	1	5	10	10	20
BRW (^°^/*h*)	0	15	30	60	90	90
Median Position Error (m)	0.81	0.92	0.93	1.32	1.63	1.86
